# Case Report: Ophthalmic endoscopy for complete resection of a symptomatic iris pigment epithelial cyst

**DOI:** 10.3389/fmed.2026.1807287

**Published:** 2026-04-30

**Authors:** Yuan Tao, Xing Du, Mengdi Wang, Chenming Zhang, Xu Wang

**Affiliations:** Department of Ophthalmology, Jinan Second People’s Hospital, Jinan, Shandong, China

**Keywords:** case report, endoscopy, iris cyst, ophthalmic endoscopy, resection, symptom

## Abstract

**Background:**

To report a case of symptomatic posterior iris pigment epithelial (IPE) cyst causing angle closure that was successfully managed with ophthalmic endoscopy.

**Case presentation:**

A female with a 1-week history of blurred vision in right eye (RE). Slit lamp and UBM examination revealed iris cysts in both eyes, and a large dominant temporal cyst in the RE produced mild forward displacement of the ciliary body and bulging of the iris root. Diagnosis was iris cyst in both eyes. Assisted by ophthalmic endoscopy, the cyst wall was removed completely without damaging the surrounding tissues or the lens. On postoperative day one, the patient’s best-corrected visual acuity (BCVA) improved, and her intraocular pressure (IOP) decreased. At the 3-months follow-up, the patient’s BCVA and IOP remained stable, and there was no cyst recurrence.

**Conclusion:**

This case suggests that endoscopic iridocystectomy is a good, viable, and minimally invasive option to treat symptomatic iris cysts.

## Introduction

Iris cysts are classified into primary cysts, arising from the iris pigment epithelial (IPE) or stroma, and secondary cysts, which can be post-traumatic (implantation), post-surgical, uveitic, or drug-induced (1, 2). There are many treatment methods; in previous case reports, Jadav and David used photocoagulation therapy with the assistance of endoscopy to treat iris cysts (3, 4), Vanita used a Phaco-endocycloplasty technique to treat cysts (5), and Sam used endophotocoagulation and vitrectomy probe to treat cysts (6), all of which achieved good results. Our report may be one of the first reports of endoscopy-assisted complete resection of a symptomatic posterior IPE cyst using intraocular forceps, while preserving the surrounding iris and crystalline lens.

## Patient information and clinical findings

**FIGURE 1:**
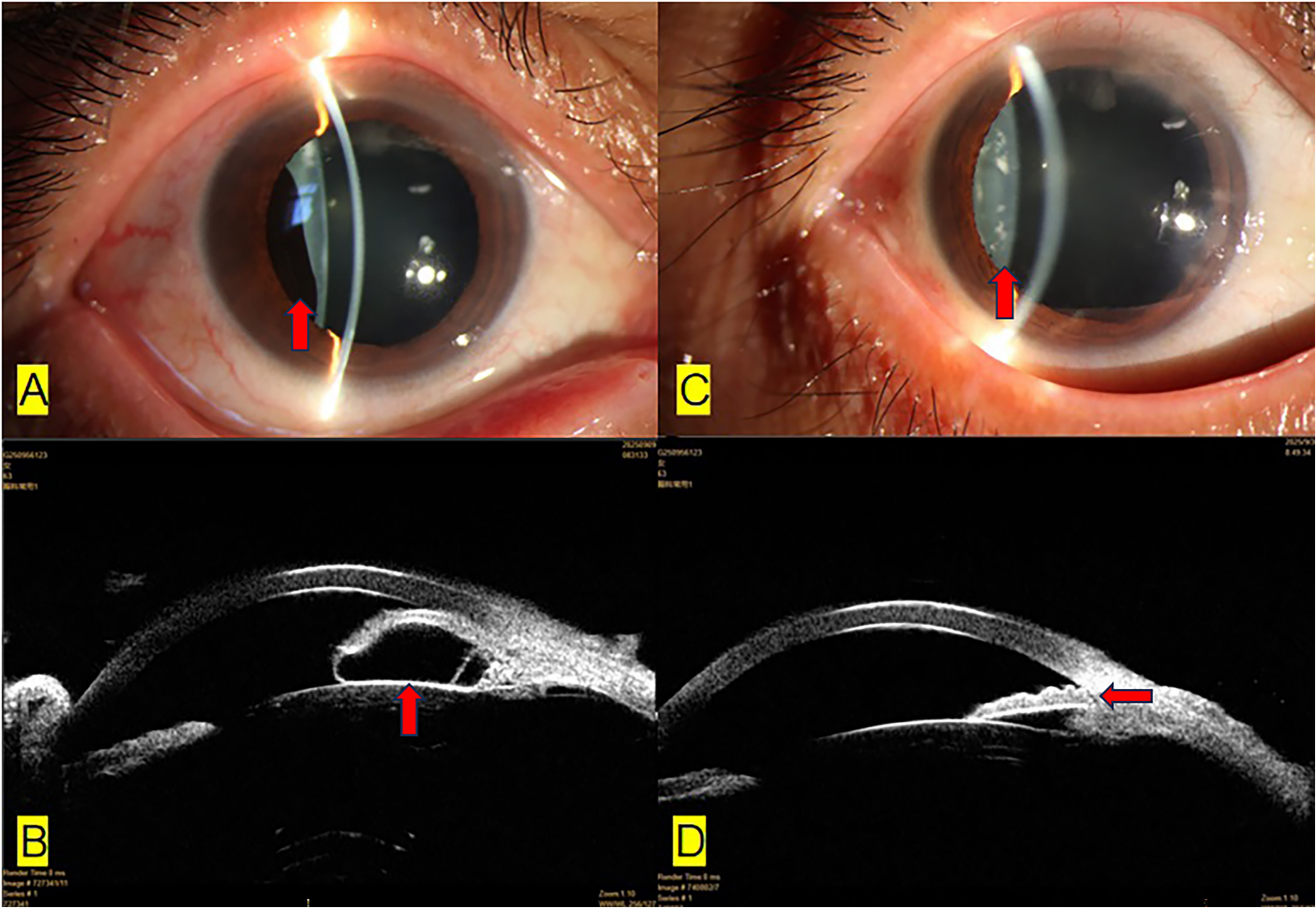
**(A)** Preoperative image of the iris cyst; after dilating the pupils, a black iris cyst can be seen behind the temporal iris of the right eye, as indicated by the red arrow. **(B)** UBM imaging of the temporal iris cyst in the right eye before surgery; iris cyst compression leads to closure of the anterior chamber angle, as indicated by the red arrow. **(C)** Postoperative images after dilating the pupils; no black iris cyst can be seen behind the temporal iris of the right eye, and there is no damage to the surrounding tissue of the iris cyst, as indicated by the red arrow. **(D)** UBM imaging of the temporal iris in the right eye; the anterior chamber angle reopened after surgery, as indicated by the red arrow.

A 63-years-old female presented on September 9, 2025, with a 1-week history of blurred vision in the right eye (RE). Best-corrected visual acuity (BCVA) was 0.15 in the RE and 0.8 in the left eye (LE). Intraocular pressure (IOP) was 20 mmHg (RE) and 10 mmHg (LE). Slit-lamp examination revealed segmental, convex bulging of the iris in both eyes, most pronounced on the temporal side of the RE (Figure 1A), which resulted in a shallow periphera anterior chamber. Both eyes showed mild cortical opacities in the lenses. Ultrasound biomicroscopy (UBM) demonstrated multiple, anechoic, oval-shaped cystic areas between the posterior iris surface and the ciliary processes in both eyes. A large dominant temporal cyst in the RE produced mild forward displacement of the ciliary body and bulging of the iris root (Figure 1B), leading to the closure of the anterior chamber angle, and a higher IOP in the RE than in the LE, with a difference in IOP between the two eyes greater than the normal value of 5 mmHg. Based on the above information, the diagnosis was iris cyst in both eyes.

## Therapeutic interventions

**FIGURE 2:**
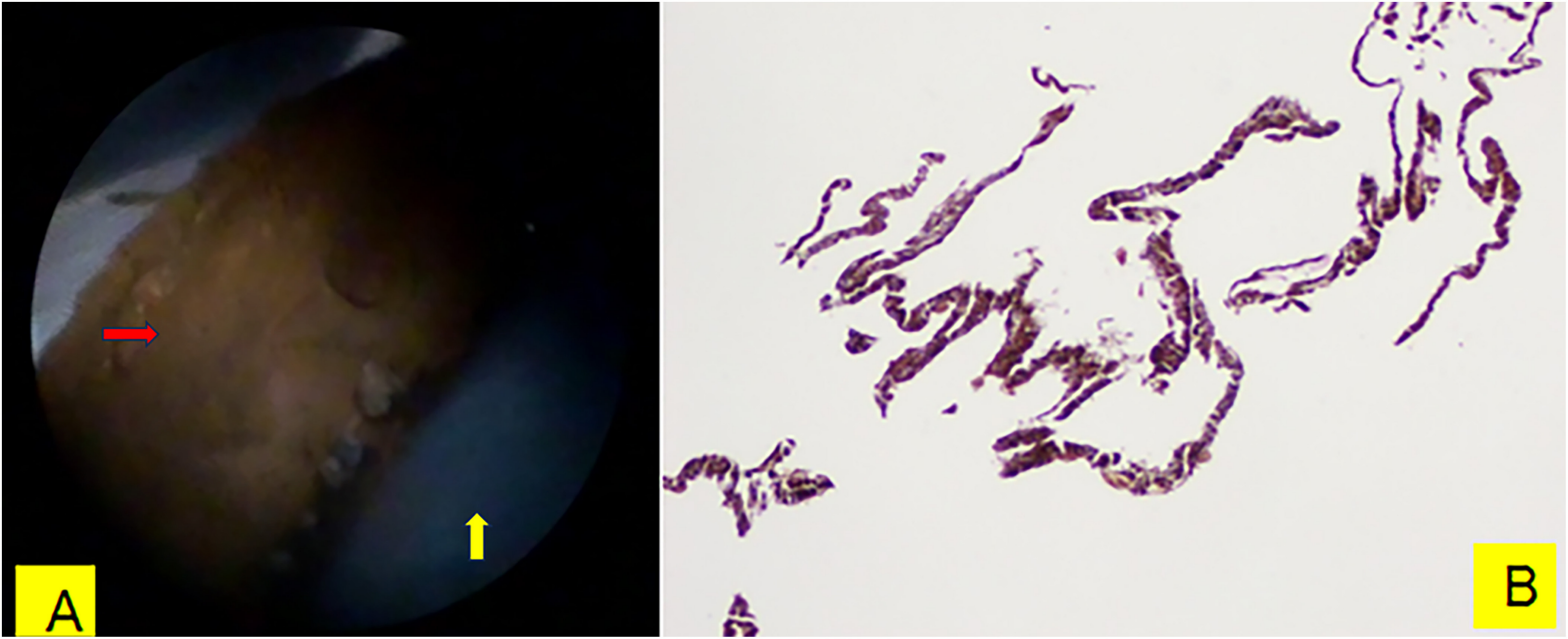
**(A)** Endoscopic image of the posterior iris surface after cyst wall was dissected, as indicated by the red arrow. The yellow arrow indicates the anterior surface of the crystalline lens. **(B)** The histopathological examination of the iris cyst confirmed the presence of IPE, and no abnormalities or tumor cells were found.

On September 10, 2025, the patient underwent an endoscopic iridocystectomy in the RE under local anesthesia. First, a 20G corneal limbal incision was made at the 5-o’clock position. Viscoelastic agent (Shanghai Qisheng Biological Preparations Co., Ltd.) was injected into the anterior chamber, and an intraocular endoscope (ENTO OPTIKS E4, USA) was introduced. A 25G corneal limbal incision was made at the 1-o’clock position to introduce a 25G intraocular forceps. Particularly, viscoelastic agent was injected behind the temporal iris to separate the iris and lens, creating surgical space for the subsequent cyst removal. With the help of the endoscope, the cyst tissue behind the temporal iris could be seen. Using the 25G intraocular forceps, the cyst wall was carefully dissected from the posterior stroma of the iris. We removed almost all of the cyst wall in the first attempt, and the remaining small amount of cyst tissue was removed in the next several steps. We didn’t open the cyst during dissection. The cyst was completely removed without damaging the surrounding tissues or the crystalline lens. During the operation, it was very important to inject an adequate amount of viscoelastic agent to avoid touching the lens, and the operation had to be performed very carefully. After removing the iris cyst, the viscoelastic agent in the anterior chamber was washed out, and the IOP was restored through the watertight corneal incision. There were no complications such as bleeding or damage to the surrounding tissues during the operation. Postoperatively, the patient received moxifloxacin eye drops (Vigamox, Alcon, USA) four times daily for 1 week and 1% prednisolone acetate eye drops (Pred Forte, Allergan, USA) four times daily for 1 week. Compound tropicamide eye drops (Mydrin-P, Santen, Japan) were administered once nightly for 1 week. During the surgery, we can see the images of the posterior iris surface after cyst wall was dissected (Figure 2A). Histopathological examination of the excised tissue confirmed the presence of IPE. No atypia or neoplastic cells were found (Figure 2B).

## Follow-up and outcomes

On the first day after surgery, the patient’s BCVA in RE improved to 0.3 and IOP decreased to 12 mmHg. Slit lamp examination confirmed the resolution of the temporal iris bulge (Figure 1C), no black iris cyst was found, the temporal anterior chamber angle had reopened, normal iris tissue was not damaged, no new opacity was observed in the lens, and there was no bleeding or exudation in the anterior chamber. During the 3-months follow-up, the patient’s BCVA and IOP remained stable, and there was no UBM evidence of cyst recurrence (Figure 1D).

## Discussion

Ultrasound biomicroscopy is currently the gold standard for diagnosing posterior-segment iris cysts, offering excellent resolution and tissue penetration to differentiate them from solid tumors and define their relationship to adjacent structures (1, 7). The decision to treat is based on the presence of symptoms or complications (1, 7), such as the visual decline and secondary angle closure seen in our patient. For symptomatic cysts, existing treatments have significant limitations. Older methods, such as cryotherapy, were largely abandoned due to complications like corneal decompensation. FNA and sclerotherapy using drug injection are plagued by high recurrence rates, as they fail to remove the secretory epithelial lining (8, 9). Laser photocoagulation is often unsuitable for IPE cysts due to their posterior location, which shields them from the laser, and the risk of pigment dispersion and IOP spikes. While sector iridectomy is definitive, the resulting structural and functional defects are often unacceptable to the patient (10). Preoperative examination showed that the anterior chamber angle of the right eye was closed at 7–10 o’clock, and there was no significant closure of the anterior chamber angle of the left eye. In order to avoid further enlargement of the temporal iris cyst, which may lead to further decline in vision and increase in IOP, a surgery was performed on the right eye. Endoscopy-assisted surgery is providing direct, high-magnification visualization of the posterior iris surface–a region that is a “blind spot” during standard anterior segment surgery. The advantages are multifaceted. First, it facilitates complete cyst wall removal, which is critical for preventing recurrence. Second, it is a tissue-sparing procedure. This method preserves the anterior iris stroma, maintaining the pupil’s integrity and preventing the functional complications of glare and photophobia. Furthermore, the procedure provides a high-quality tissue specimen for histopathological analysis. This treatment method also has the following drawbacks: (1) Operations in the anterior chamber may damage surrounding tissues, such as damaging the lens causing cataracts and removing the normal iris causing iris damage. (2) Endoscopic intraocular surgery requires high technical skills from the surgeons. Because it is different from conventional ophthalmic surgery where various operations are performed under direct vision, endoscopic surgery requires both direct vision and continuous monitoring of the display screen to clarify the images under the endoscope. Besides, at present, the follow-up time is relatively short, and we will continue to observe to clarify the long-term effects.

## Conclusion

This case suggests that endoscopic iridocystectomy is a good, viable, and minimally invasive option to treat symptomatic iris cysts. This method deserves further research, as longer follow-up and additional cases are required before broader conclusions can be drawn.

## Data Availability

The original contributions presented in this study are included in this article/supplementary material, further inquiries can be directed to the corresponding author.
